# Serotonergic control of feeding microstructure in *Drosophila*

**DOI:** 10.3389/fnbeh.2022.1105579

**Published:** 2023-01-17

**Authors:** Ayesha Banu, Swetha B. M. Gowda, Safa Salim, Farhan Mohammad

**Affiliations:** Division of Biological and Biomedical Sciences (BBS), College of Health and Life Sciences (CHLS), Hamad Bin Khalifa University (HBKU), Doha, Qatar

**Keywords:** serotonin, *Drosophila*, 5-HT receptor, feeding microstructure, hunger

## Abstract

To survive, animals maintain energy homeostasis by seeking out food. Compared to freely feeding animals, food-deprived animals may choose different strategies to balance both energy and nutrition demands, per the metabolic state of the animal. Serotonin mediates internal states, modifies existing neural circuits, and regulates animal feeding behavior, including in humans and fruit flies. However, an in-depth study on the neuromodulatory effects of serotonin on feeding microstructure has been held back for several technical reasons. Firstly, most feeding assays lack the precision of manipulating neuronal activity only when animals start feeding, which does not separate neuronal effects on feeding from foraging and locomotion. Secondly, despite the availability of optogenetic tools, feeding in adult fruit flies has primarily been studied using thermogenetic systems, which are confounded with heat. Thirdly, most feeding assays have used food intake as a measurement, which has a low temporal resolution to dissect feeding at the microstructure level. To circumvent these problems, we utilized OptoPAD assay, which provides the precision of optogenetics to control neural activity contingent on the ongoing feeding behavior. We show that manipulating the serotonin circuit optogenetically affects multiple feeding parameters state-dependently. Food-deprived flies with optogenetically activated and suppressed serotonin systems feed with shorter and longer sip durations and longer and shorter inter-sip intervals, respectively. We further show that serotonin suppresses and enhances feeding *via* 5-HT1B and 5-HT7 receptors, respectively.

## 1. Introduction

Feeding is a fundamental and defining behavior of heterotrophic organisms for energy homeostasis and survival. Its regulation is an essential aspect of an animal’s fitness (Morton et al., 2014) and psychological wellbeing, as dysfunctional feeding may lead to feeding and eating disorders (DSM-5, Roehr, 2013)–an increasing health burden worldwide (Treasure et al., 2020).

Many aspects of feeding are conserved between mammals and insects (Yellman et al., 1997). Using *Drosophila* as a model system, substantial progress has been achieved in understanding the molecular and neuronal mechanisms regulating feeding (Itskov and Ribeiro, 2013). Similar to vertebrates, feeding or consumption of food in *Drosophila* comprises various sensory, cognitive, ingestive, post-ingestive, and post-absorptive characteristics (Branch and Shen, 2017) and depends on multiple variables like animal energy demand (Pool and Scott, 2014), food quality, and palatability (Yeomans, 1998), nutrient value and caloric content (Dus et al., 2011; Itskov and Ribeiro, 2013; Ro et al., 2016), and food texture (Sanchez-Alcaniz et al., 2017). Together, these mechanisms determine *Drosophila*’s decisions and affect food consumption. Food-deprived flies enhance their food intake to compensate for reduced energy and nutrients homeostatically. Eating too much or too little could have adverse effects, so the desire to eat and satiety must be equally regulated (Schoener, 1971). Flies may also use distinct strategies depending upon the length of food deprivation and the nature and characteristics of food. The important ways to alter feeding in food-deprived flies could be changing the feeding rate, duration of feeding events, and food preferences (Lin et al., 2019). Flies’ feeding strategies in short to medium food deprivation (4–16 h) time have been studied (Itskov et al., 2014), but the effects of prolonged food deprivation have focused on changes in metabolism and the mechanisms of development of starvation resistance. How extended food deprivation affects feeding strategies, and the regulation of feeding needs to be explored at a deeper level.

Like humans and rodents, flies also exhibit highly rhythmic feeding patterns (Moulin et al., 2021) in which every interaction with food consists of many temporally distributed fundamental quantitative units of ingestive behavior for which the numbers, durations, and intervals can be defined and are together known as the microstructure of feeding (Davis, 1989). Units within a microstructure are interdependent—for example, increased sip duration (meal size) results in decreased intervals between sips (meal rate). In rats, longer pauses between sips or larger inter-sip intervals reflect the integration of ingestive neural signals (Smith, 2001). To maintain energy balance, an organism must regulate the microstructure of feeding by integrating peripheral signals with internal states (Gillette, 2006). Dysfunction in the complex interaction between energy demand and feeding behavior has detrimental effects on human physiology. It could lead to obesity, diabetes, addiction, and associated life-threatening diseases (Llewellyn et al., 2008; Morton et al., 2014; Langlet et al., 2017). Characterizing neurons and neural circuits controlling individual units of feeding microstructure will aid in a deeper understanding of homeostasis and the study of mechanisms by which feeding behavior can become maladaptive, leading to the pathology of feeding and eating disorders. Apart from being valuable models for understanding the genetic, molecular, and neuronal mechanisms of feeding, understanding insects’ feeding has more direct and profound medical, ecological, and economic implications, as many insect species are medical and horticultural pests. Understanding their feeding patterns can help combat them (Scott and Takken, 2012).

Serotonin, a highly conserved monoamine across phylogeny, represents the internal states of the animals and orchestrates both physiological and behavioral determinants of energy balance (Gillette, 2006; Tecott, 2007). Its role in feeding has been the focus of much research in the past few decades in both invertebrates and vertebrates. In invertebrates, it has been shown to control both hunger and satiety, and systemic serotonergic manipulation in different taxonomic groups have yielded different outcomes, and it was shown to modulate specific aspects of feeding in different model systems (Tierney, 2020). In mammals, serotonin is known to affect satiety and is generally thought of as a feeding suppressant and has been the target of many anti-obesity drugs (Donovan and Tecott, 2013; Voigt and Fink, 2015; Yabut et al., 2019). However, serotonin is known to play a role in both food ingestion (Plassmann et al., 2022) and locomotion (Flaive et al., 2020), and changes in the locomotion could obscure changes in the feeding pattern. Therefore, it is imperative to dissociate locomotor aspects from feeding behavior.

Feeding analysis has mostly been performed using quantitative food intake measurement (Albin et al., 2015; Pooryasin and Fiala, 2015). Most assays measuring food intake are low in precision to quantify feeding patterns at the microstructure level; however, with advancement, a few methods have been developed using which food consumption can be quantitatively measured at the microstructure level (Itskov et al., 2014; Yapici et al., 2016). Using these high-resolution feeding monitoring systems, the role of IN1 interneurons, which receive sweet input from the pharyngeal sense organs, has been revealed in regulating ingestion of a sweet solution by controlling the volume per bout and the rate of drinking (Yapici et al., 2016). Although neuromodulators like serotonin or dopamine are known to alter food ingestion by modulating the neuronal activity of sensory neurons (Inagaki et al., 2014; Albin et al., 2015), to our knowledge, no central neuromodulatory neurons have been implicated in controlling specific aspects of the microstructure of feeding in any model system so far.

Despite the known role of broad and systemic serotonin in affecting feeding behavior in multiple organisms (Blundell, 1992), serotonergic pathways which regulate specific aspects of microstructure of feeding behaviors are not known. There are several reasons why the in-depth study on the neuromodulatory effects of serotonin on feeding microstructure has been held back. Most neuronal manipulation starts way before animals start feeding, precluding differentiation between effects on motivation and locomotion. The neuronal manipulations used so far are either based on pharmacological or thermogenetic activations (Albin et al., 2015; Pooryasin and Fiala, 2015), which either lack specificity or are confounded with effects like heat, which is not only in the aversive range to the fly but may also promote feeding (Klepsatel et al., 2019). Moreover, neurons activated through the thermogenetic system may show spike decay within seconds (Inagaki et al., 2014), suggesting that thermogenetic manipulations may cause adaptation in some neurons in longer activation experiments. Optogenetics-based activation strategies are faster and more precise. They can also be paired with ongoing feeding behaviors, and the direct effects of neuronal activity on feeding behavior can be studied.

In this study, we tested the fly’s feeding microstructure using the OptoPAD system (Moreira et al., 2019), which logs fly sipping events and, through a feedback loop, controls LED illumination contingent upon fly feeding behavior, allowing assessment of optogenetics-mediated neuronal activity effects only on ongoing feeding behavior and dissociating it from serotonin’s effects on locomotion. We expressed optogenetic activator CsChrimson (Klapoetke et al., 2014) or optogenetic inhibitor GtACR1 (Mohammad et al., 2017) in serotonergic systems using the Gal4/UAS system. We utilized the genetic intersection approach to study the role of serotonergic neurons in the brain and VNC and multiple serotonin receptor alleles to delineate the neuronal pathway controlling feeding microstructure.

## 2. Materials and methods

### 2.1. Fly stocks and medium

All fly strains were maintained at a temperature of 25°C and 70% humidity with a 12 h light-dark cycle in Darwin Chambers (IN084-AA-LT-DA-MP). All fly strains were reared on Nutri-Fly Bloomington Formulation food medium (Genesee Scientific, Cat #66-116). Food was prepared according to the package instructions using 177 g of media powder per liter of distilled water. After cooling to 70°C, 4.9 mL of preservative Propionic Acid (Merck-Sigma, C38006052500) and 10 ml of 10% antifungal Nipagin (Tegosept, Genesee Scientific, cat #20-259) dissolved in absolute ethanol was added per liter of food.

### 2.2. Fly stocks for behavioral experiments

Genotypes of the fly lines used in the experiments are listed in Supplementary Tables 1, 2. F1 generation male flies were used for all behavioral experiments and maintained in Darwin Chambers (IN084-AA-LT-DA-MP) at a temperature of 25°C and 70% humidity with a 12 h light-dark cycle. To assay in a sated state, flies were directly transferred from the food medium to the assay set-up. For food deprivation, the flies were wet starved in vials containing 0.8% agarose for 24–27 h before assaying.

### 2.3. Optogenetic experiments

Chrimson and GtACR1 expressing flies used for optogenetic experiments were transferred 0–3 days after eclosion to a medium containing all-trans-retinal (1 mM ATR, Carbosynth, #16-31-4) prepared in 100% ethanol with minimal exposure to light. Flies were reared on ATR mixed medium for at least 48 h before further experimentation. The light intensity was measured for red and green illumination for wavelengths (λ635 and λ532 nm) using the optical power meter (Thorlabs PM400) and optical sensor (Thorlabs S120C).

### 2.4. FlyPAD assay

FlyPAD arenas (V2, 2018, Easy Behavior)^1^ were used for all the experiments. All FlyPAD food ports were loaded with approx. 5 μL of 5 mM sucrose (prepared in 0.8% Agarose) in each well in all the experiments, except where mentioned otherwise. For the assay, 3–7 days old male flies were briefly anesthetized on ice and loaded into the FlyPAD with one fly per arena. The assay duration was 1 h, and all experiments were conducted in an incubator at 25°C (PHCbi, MIR-154-PE). Capacitance files were saved locally and analyzed. The conditions and event labels were recorded in a. txt file for each set of experiments.

### 2.5. OptoPAD assay

All FlyPAD food ports were loaded with approx. 5 μL of 5 mM sucrose (prepared in 0.8% Agarose) in each well in all experiments, except where mentioned otherwise. For the assay, 3–7 days old male flies reared on ATR media were briefly anesthetized on ice and loaded into the arenas quickly to minimize light exposure. The OptoPAD LEDs were connected to each arena (Easy Behavior)^1^. The assay was run for 1 h in an incubator at 25°C (PHCbi, MIR-154-PE) with minimal light from outside. The assay was conducted using Bonsai software real-time analysis. Red illumination of 35 and 140 μW/mm^2^ and green illumination at 20 or 100 μW/mm^2^ were achieved using a power supply (0–5 V). Thresholds for the OptoPAD devices were set at 150. The light was switched on in the closed-loop protocol starting with fly contact with food (0 s) and remaining on for 2 s irrespective of fly behavior. Flies of the same genotype assayed in this setup but without LED illumination were used as controls. Capacitance files were saved locally and analyzed. The conditions and substrate labels were recorded in a. txt file for each set of experiments.

### 2.6. Microstructure analysis

Feeding microstructure analysis was performed as described earlier (Itskov et al., 2014), using software written in the Matlab runtime engine to analyze all the capacitance data (Runme_Mean_29_04_2021_v2_5_MergeChannelsNEW). Output data saved in excel format was used to generate scatter plots using the EstimationStats.com web application.

### 2.7. Statistical analysis

The *P*-values were calculated by permutation *t*-test using 5,000 bootstrap sampling. For each *p*-value, 5,000 reshuffles of the control and test labels were performed Estimation Stats web application) (Ho et al., 2019). The Shapiro–Wilk normality test (Statskingdom web application) was used to assess whether the data followed a normal distribution. Cohen’s *d* was used as the effect size measure for data following a Gaussian distribution. For non-Gaussian distributed data, effect sizes were calculated using Cliff’s Δ. All heat maps are shown with Cliff’s Δ effect sizes. The sip duration parameter followed a normal distribution pattern in almost all the datasets. Hence, Cohen’s *d* effect sizes are reported for sip duration plots.

### 2.8. Proboscis extension reflex (PER) assay

F1 generation 3–5-day old male flies were starved for 20–22 h, and individual flies were glued on their back on the thorax using an odorless glue on a 40 mm × 60 mm coverslip. The flies were allowed to recover in a humidified chamber for 2–3 h. The coverslips were placed on the microscope stage using paper clips as holders in such an orientation that they were being viewed laterally. Flies were illuminated with red light from a projector (OPTOMA, ML750) placed 14 cm away to test their PER response. Using a 1 mL syringe, the fly was stimulated on its tarsi for the different tastants (water, 5, 50, and 500 mM sucrose) three times for each. The video was recorded in the Leica software and scored for PER response manually. Flies from the same batch without illumination were used as sibling controls. The response was recorded as 0 for no extension and 1 for any number of extensions within the three stimulations. PER percentage and *p*-value for the student’s *t*-test were calculated, and a line plot was generated in Excel.

### 2.9. Immunohistochemistry and confocal microscopy

The fly brains were dissected in isotonic PBS (Phosphate Buffer Saline) and fixed in 4% paraformaldehyde (PFA) for 20 min at room temperature. The samples were then washed with PBST (PBS with 1% Triton X-100) to remove the fixative completely, two quick washes followed by four washes at 15 min intervals. The samples were then blocked for 30 min using a blocking solution (PBST with a 1% BSA) and incubated with primary antibodies [Mouse anti-Dlg (1:50), Developmental Studies Hybridoma Bank- DSHB, RRID:AB_528203] or [Rat anti-5-HT (1:50), Merck-Millipore, RRID:MAB-352] overnight at 4°C on a shaker at 50 rpm. The next day, samples were washed with PBST four times at 15 min intervals and incubated with secondary antibodies for 3–4 h at room temperature. [Goat anti-Mouse 568 (1:200), Rabbit anti-Rat 594 (1:200) or Goat anti-Rat 488 (1:200), Thermo Fisher Scientific-Invitrogen]. Then, samples were washed with PBST four times at 15 min intervals. The processed brain samples were then mounted onto a glass slide using Vectashield mounting medium (Vector Laboratories). Confocal images were obtained using a Nikon A1 confocal microscope. Images were analyzed in ImageJ and presented as Maximum Intensity Projection (MIP).

## 3. Results

### 3.1. Food deprivation induces changes in feeding strategies in wild-type flies

**FIGURE 1:**
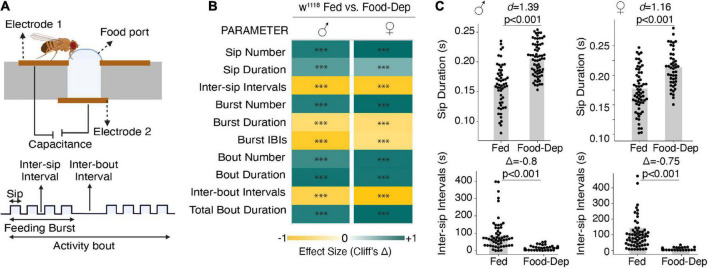
Prolonged food deprivation induces feeding by modulating feeding microstructure. **(A)** Schematic depicting the FlyPAD setup and the feeding microstructure parameters measured in fed and 24-h food-deprived flies. **(B)** Heat maps showing the effect sizes (Cliff’s Δ) between fed and food-deprived male and female flies for the ten feeding parameters measured using FlyPAD. **(C)** Cumming estimation plots of the sip duration and inter-sip intervals. Food-deprived flies show an increase in sip duration compared with fed flies during the assay (3,600 s) in both males [*n* = 54–67, *d* = 1.39 (95 CI 0.97, 1.8), *p* < 0.001] and females [*n* = 52–61, *d* = 1.16 (95 CI 0.759, 1.54), *p* < 0.001]. Food-deprived flies show a decrease in inter-sip intervals compared with fed flies during the assay (3,600 s) in both males [*n* = 55–65, Δ = –0.822 (95 CI –0.92, –0.65), *p* < 0.001] and females [*n* = 52–61, Δ = –0.75 (95 CI –0.89, –0.56), *p* < 0.001] In the heatmap, green indicates an increase in effect size, and yellow indicates a decrease in effect size in food-deprived compared to freely feeding flies. *p*-values for the effect size measure are indicated with an asterisk *p < 0.05, ^**^p < 0.01, and ^***^p < 0.001.

We first studied male and female wild-type flies that were food-deprived for longer durations (24–27 h) to observe changes in their feeding pattern, which can be modulated by altering the number and/or duration of feeding events or varying the intervals between feeding events. We used the FlyPAD, a capacitance-based system, to measure fly-feeding behavior in a detailed, high-throughput manner (Itskov et al., 2014). FlyPAD system measures the interaction with food and defines the feeding microstructure in terms of sips, feeding bursts (meals), and activity bouts (overall interactions with food) (Figure 1A).

Compared to freely feeding or sated flies, 24-h food-deprived flies exhibited a many-fold change in all feeding parameters in both male and female flies (Figure 1B). For example, in 1 h of assay time, the sip duration was significantly longer, and inter-sip intervals were shorter in 24-h food-deprived flies (Figure 1C). Overall, in 24-h food-deprived flies, there were 500 times more sips, 50 times more feeding bursts, and 120 times more activity bouts (Figure 1B) than in fed flies. Like the enhanced number of feeding events, food-deprived flies also exhibited an enhanced feeding rate as prolonged deprivation showed reduced inter-sip intervals (Figure 1C), inter-burst intervals (IBIs), and activity-bout intervals (Figure 1B). We also measured the length or duration of every feeding event. Interestingly, sip duration and activity bout duration were significantly higher in 24-h food-deprived flies (Figures 1B, C). However, the feeding burst size was considerably reduced (Figure 1B).

Taken together, our data suggest that following longer food deprivation, flies adjust their feeding microstructure by enhancing the feeding rate and have much more frequent interactions with food but reduce the duration of feeding bursts, suggesting that flies food-deprived for long duration use a strategy of many meals but each of short duration. This may allow a severely food-deprived fly to eat from many food sources before attaining satiety. We didn’t detect any difference in feeding patterns between male and female flies, so we only used male flies in all further experiments.

### 3.2. Activity in a broad serotonergic circuit induces state-dependent effects on feeding microstructure

Although molecular mechanisms controlling physiology and metabolism in flies are largely conserved with vertebrates (Bharucha, 2009), it needs to be clarified if neuromodulatory control of the feeding-related behavioral strategy (microstructure of feeding) is similarly conserved. To study whether and how serotonin modulates hunger and satiety in flies and whether it modulates a specific aspect of feeding, we assayed freely feeding (sated) and food-deprived flies using the OptoPAD (Moreira et al., 2019). OptoPAD is a system that allows the optogenetic manipulation of circuit activity in *Drosophila* conditionally, depending on ongoing feeding behavior, using a closed-loop system.

**FIGURE 2:**
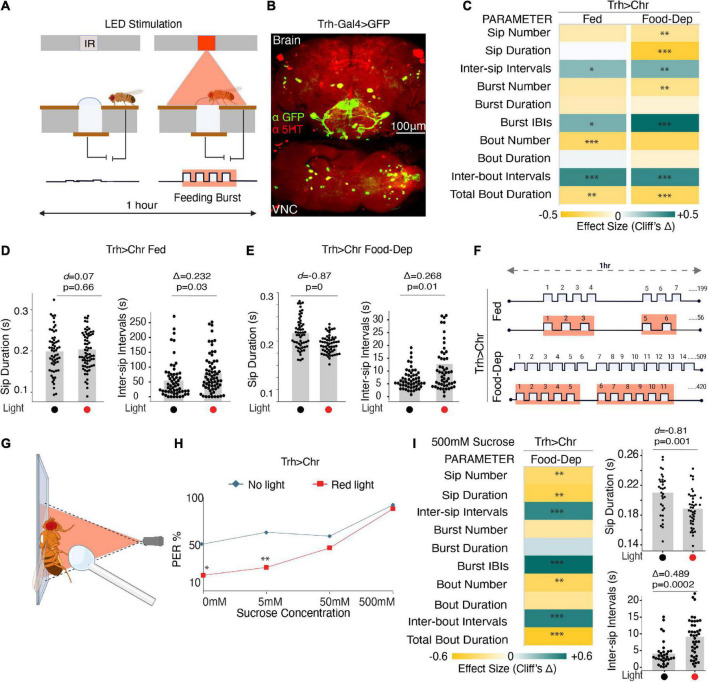
Optogenetic activity in the broad serotonin system suppresses feeding. **(A)** Schematic depicts the closed-loop OptoPAD system used for optogenetic activation of serotonergic neurons as the fly starts to feed. The light stimulation is provided for 2 s after the start of an activity bout. **(B)** Maximum intensity projections of Trh-Gal4 driven expression of mcD8:GFP and immunostained with anti-5HT antibody to mark the serotonergic neurons in the fly brain and VNC. **(C)** Heat map showing Cliff’s Δ effect size for all the parameters measured by the OptoPAD system for *Trh-Gal4* > *UAS-Chrimson* in the fed (left) and the food-deprived state (right). **(D)** Estimation plot showing Cohen’s *d* for mean sip durations and Cliff’s Δ for inter-sip intervals for fed *Trh-Gal4* > *UAS-Chrimson* flies with and without optogenetic activation during the assay time (3,600 s) (*n* = 60–70). Optogenetic activation of Trh-expressing neurons increased the inter-sip intervals [*n* = 57–65, Δ = 0.232 (95% CI 0.026, 0.422), *p* = 0.03]. **(E)** Estimation plot showing the Cohen’s *d* for mean sip durations and inter-sip intervals of food-deprived *Trh-Gal4* > *UAS-Chrimson* flies with and without optogenetic activation during the assay time (3,600 s). Activation of Trh-expressing neurons reduced sip duration [*n* = 56–57, *d* = –0.87, (95% CI, –1.23, –0.52), *p* < 0.001] and increased the inter-sip intervals [*n* = 55–56, Δ = 0.268 (95% CI 0.04, 0.47), *p* = 0.01]. **(F)** Representative schematic comparing the changes in feeding microstructure parameters with the optogenetic activation of *Trh-Gal4* > *UAS-Chrimson* flies compared with controls without activation for both fed and food-deprived states. An average number of sips is shown; sip durations, and intervals indicate changes not drawn to scale. **(G)** Schematic depicting the PER assay with optogenetic manipulation. **(H)** PER response in red light illuminated *Trh-Gal4* > *UAS-Chrimson* flies is decreased when presented with water (0 mM sucrose) (*n* = 24, *p* = 0.014) and 5 mM sucrose (*n* = 24, *p* = 0.008) compared to non-light activated controls. **(I)** (Left) Heat map showing Cliff’s Δ effect size for all the parameters measured by the OptoPAD system using 500 mM sucrose as the food source. (Right) Estimation plots show Cohen’s *d* for sip duration and Cliff’s Δ for inter-sip intervals for 500 mM sucrose. There is a significant reduction in sip durations [*n* = 32–42, *d* = –0.81 (95% CI –1.34, –0.27), *p* = 0.001] and an increase in intervals between them [*n* = 32–41, Δ = 0.489 (95% CI 0.201, 0.683), *p* = 0.0002]. All optogenetic activation experiments were performed at 140 μW/mm^2^. In the heatmap, green indicates an increase in effect size, and yellow indicates a decrease in effect size. *p*-values for the effect size measure are indicated with an asterisk *p < 0.05, ^**^p < 0.01, and ^***^p < 0.001.

We used flies expressing CsChrimson in the Trh-Gal4 line, representing almost 80% of serotonergic cells (Alekseyenko et al., 2010; Raghu et al., 2018) (Figure 2B), and assayed them in two conditions–fed and food-deprived. Fed flies had access to food *ad libitum* and approached the food in a normal hunger state. Food-deprived flies were starved on moist agarose for 23–27 h before assaying. We compared these CsChrimson-expressing flies with and without red illumination (λ625 nm) (Figure 2A). Light activation was set to 0 s after each activity-bout started and sustained for 2 s, irrespective of fly behavior (Moreira et al., 2019). The flies were assayed at two different red illumination levels (∼35 μW/mm^2^ and ∼140 μW/mm^2^) (Moreira et al., 2019).

At lower illumination (∼35 μW/mm^2^), there was no significant difference in any of the feeding parameters in the fed state, but in the food-deprived state, a small increase in inter-sip intervals and a decrease in bout duration was observed (Supplementary Figures 1A, B), however, the changes in feeding patterns were statistically significant at higher red illumination (140 μW/mm^2^) suggesting that a higher irradiance was required for the proper functioning of the ion channels and subsequent activation of these neurons. Subsequently, higher illumination was used in all chrimson activation experiments.

In the fed state, activating serotonergic cells when flies start feeding significantly reduced the number of feeding bouts and total activity bout durations, along with an increase in all the interval parameters (Figures 2C, F), suggesting a decrease in feeding rate and general suppression of feeding. At the level of sips, durations were unaffected, but intervals between them increased (Figure 2D). In contrast, optogenetic activity in the broad serotonergic circuit in food-deprived flies reduced the number, durations, and rate of all feeding parameters (Figures 2C, E). The mean sip duration was significantly decreased, and intervals were increased in the case of food-deprived flies (Figures 2C, E). Overall, our data suggest that serotonin in feeding flies suppresses feeding both in fed and food-deprived states. While freely feeding flies (sated) with enhanced serotonin exhibit reduced feeding rates (longer IBIs), food-deprived flies, despite the strong motivation to feed, with activated serotonin exhibit significant suppression of complete feeding microstructure (Figures 2C, F).

To rule out any effects of light illumination on the feeding assay, and as genotypic controls, we compared age-matched and retinal-treated isogenic *w*^1118^ wild-type flies, *Chrimson > w*^1118^ flies, and *Trh > w*^1118^ flies with and without red illumination (Supplementary Figures 1C–E) in both the freely fed and food-deprived state. There was no significant change in the majority of the parameters except for the slight changes in total food interaction times or the number of some of the feeding parameters when illuminated in the fed state (Supplementary Figures 1C–E), which could be explained by the non-uniformity of the hunger states in the fed flies and perhaps a slight feeding promoting effect from the perception of light. There was no significant change in any parameter in the food-deprived states for any of these controls.

Since serotonergic activation had a stronger and more consistent effect in the food-deprived state, to confirm if the observed pattern of feeding microstructure was indeed driven by serotonin, we used Trh-RNAi to deplete serotonin in the same cells driven by Trh-Gal4 with concurrent chrimson based neuronal activation (Supplementary Figure 1F). In the case of broad serotonin depletion in the Trh-Gal4 cells, all effects observed earlier when serotonin was optogenetically activated were eliminated, except for a slight increase in intervals between activity bouts (Supplementary Figures 1G, H), confirming that the feeding pattern observed upon activation of Trh-Gal4 cells was indeed due to serotonergic activity.

To further study if serotonin-induced suppression in feeding is occurring through changes in feeding initiation, we tested the flies’ responsiveness to water and increasing concentrations of sucrose by Proboscis Extension Reflex (PER) assay (Pooryasin and Fiala, 2015) modified for use with optogenetics (Figure 2G). There was a significant reduction in the flies’ response toward the water and 5 mM sucrose when activated with red light compared to non-light activated controls (Figure 2H). Further, the responsiveness of serotonin-activated flies shifted toward high sucrose concentrations (Figure 2H). Since there was no difference in the PER between the experiment and controls at 500 mM sucrose, we tested feeding with the 500 mM sucrose on the OptoPAD to see if the feeding pattern remained comparable to 5 mM sucrose. And indeed, serotonin activation suppresses feeding and modulates the pattern of the sips even when the flies are provided with highly palatable 500 mM sucrose (Figure 2I).

### 3.3. Brain serotonin suppresses feeding in a hunger-state-dependent manner

**FIGURE 3:**
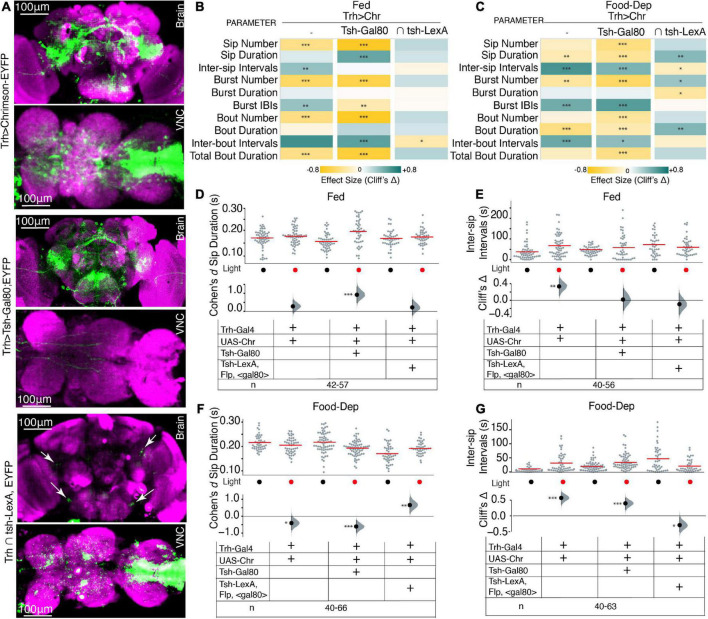
Serotonin in the brain and VNC modulates feeding microstructure in a state-dependant manner. **(A)** Maximum intensity projections of expression patterns of serotonergic neurons in both brain and VNC labeled by *Trh-Gal4* > *UAS-Chrimson-YFP* (upper), the brain only labeled by *Trh-Gal4* ∩ *tsh-Gal80;UAS-Chrimson-YFP* (middle) and VNC only in *Trh* ∩ *tub* > *gal80* > *tsh-LexA,LexAop-Flp;Chrimson-YFP* (lower); all immunostained with anti-dlg antibody. The scale bar is 100 um. White arrows in lower panels indicate positions of serotonergic PLP and SEL neurons. **(B)** Heat map showing Cliff’s Δ effect sizes for all the parameters measured by the OptoPAD system for *Trh* > *Chrimson,Trh* ∩ *tsh-gal80;Chrimson*,and *Trh* ∩ *tub* > *gal80* >*; tsh-LexA,LexAop-Flp;Chrimson* for the fed state. **(C)** Heat map showing Cliff’s Δ effect size for all the parameters measured by the OptoPAD system for *Trh* > *Chrimson, Trh* ∩ *tsh-gal80;Chrimson*, and *Trh* ∩ *tub* > *gal80* >*; tsh-LexA,LexAop-Flp;Chrimson* for the food-deprived state. **(D)** Cumming estimation plot showing the Cohen’s *d* for mean sip durations in a fed state for *Trh* > *Chrimson* flies with and without optogenetic activation during the assay time (3,600 s) compared with *Trh* ∩ *tsh-gal80;Chrimson*, and *Trh* ∩ *tub* > *gal80* >*; tsh-LexA,LexAop-Flp;Chrimson.* Optogenetic activation of serotonergic neurons in the VNC shows a significant increase in sip durations [*Trh* ∩ *tsh-gal80; Chrimson, d* = 0.921 (95.0% CI, 0.46, 1.37), *p* = 0.0]. **(E)** Cumming estimation plot showing Cliff’s Δ for inter-sip intervals in the fed state for *Trh* > *Chrimson* flies with and without optogenetic activation during the assay time (3,600 s) compared with *Trh* ∩ *tsh-gal80;Chrimson*, and *Trh* ∩ *tub* > *gal80* >*; tsh-LexA,LexAop-Flp;Chrimson.* The inter-sip interval is increased only in the broad serotonergic activation [*Trh* > *Chr n* = 55–56, Δ = 0.335 (95.0% CI 0.11, 0.52), *p* = 0.002]. **(F)** Cumming estimation plot showing the Cohen’s *d* for mean sip durations in a food-deprived state for *Trh* > *Chrimson* flies with and without optogenetic activation during the assay time (3,600 s) compared with *Trh* ∩ *tsh-gal80;Chrimson*, and *Trh* ∩ *tub* > *gal80* >*; tsh-LexA,LexAop-Flp;Chrimson.* There is a significant decrease in sip duration in broad serotonergic activation [*Trh* > *Chr n* = 44–46, *d* = –0.506 (95.0% CI –0.89, –0.1), *p* = 0.0] and even with activation of only the brain serotonergic neurons [*Trh* ∩ *tsh-gal80;Chrimson n* = 66, *d* = –0.609 (95.0% CI –0.96, –0.24) *p* = 0.0006] but there is a significant increase in sip duration when activation is limited only to the VNC [*Trh* ∩ *tub* > *gal80* >*; tsh-LexA,LexAop-Flp;Chrimson, n* = 40–44, *d* = 0.658 (95.0% CI 0.17, 1.1) *p* = 0.0052]. **(G)** Cumming estimation plot showing Cliff’s Δ for inter-sip intervals in the food-deprived state for *Trh* > *Chrimson* flies with and without optogenetic activation during the assay time (3,600 s) compared with *Trh* ∩ *tsh-gal80;Chrimson*, and *Trh* ∩ *tub* > *gal80* >*; tsh-LexA,LexAop-Flp;Chrimson*. There is a significant increase in inter-sip intervals in broad serotonergic activation [*Trh* > *Chr n* = 43–49, Δ = 0.568 (95.0% CI 0.357, 0.734), *p* < 0.0001], and even with activation of only the brain serotonergic neurons [*Trh* ∩ *tsh-gal80;Chrimso, n* = 61–63, Δ = 0.396 (95.0% CI 0.2, 0.57) *p* = 0.0002] but there is a significant decrease in inter-sip intervals when the activation is limited only to the VNC [*Trh* ∩ *tub* > *gal80* >*; tsh-LexA,LexAop-Flp;Chrimson n* = 40–43, Δ = –0.293 (95.0% CI –0.51, –0.03) *p* = 0.0214]. In the heatmap, green indicates an increase in effect size, and yellow indicates a decrease in effect size. *p*-values for the effect size measure are indicated with an asterisk *p < 0.05, ^**^p < 0.01, and ^***^p < 0.001.

Next, we asked if the neurons that are regulating this feeding microstructure are in the brain or in the VNC; we used an intersectional genetics approach to limit the activation of serotonergic cells to either the brain or the VNC. Using *Tsh-Gal80*, expression of *Trh-Gal4* was restricted only to the brain, and with intersection using the flippase base system (*tub > Gal80*; *tsh-LexA, LexAop-Flp*), Trh-Gal4 expression was restricted to the VNC (here on labeled as *Trh* ∩ tsh), which allowed activation of serotonin in the VNC only (Figure 3A).

Interestingly, upon removing Trh expression from the VNC and only activating brain serotonergic neurons, most feeding parameters were recapitulated in the same direction, similar to Trh neuron activation in the whole CNS, and effects on various feeding parameters were enhanced in both freely feeding and food-deprived states (Figures 3B, C) compared to non-light activated controls. Surprisingly, in fed flies with restricted serotonin expression to the brain, the fly’s sip durations were enhanced; in contrast, the inter-sip intervals were increased only when all CNS serotonin neurons were activated (Figures 3B, C). These experiments suggest that VNC serotonergic neurons play some role in the fed state. Identifying those serotonergic neurons in VNC would be crucial in implicating VNC in regulating some aspects of feeding microstructure; however, currently available genetic tools do not allow for the selection and dissection of the role of individual VNC serotonergic neurons.

Next, we utilized the intersection of Tsh-LexA, and Trh-Gal4 (by genetically crossing Trh-Gal4 with *tub > Gal80 > tsh-LexA, LexAop-Flp:Chrimson*) to restrict Trh-Gal4 expression to VNC. Most of the Trh expression from the brain was eliminated; however, expression in a few posterior lateral protocerebrum (PLP) and lateral subesophageal ganglia (SEL) neurons was observed (Figure 3A, lower panels, white arrows). From their position, the SEL neurons appear to be sugar-SELs (Yao and Scott, 2022). Upon activation of these cells, there was no significant change in almost any parameters in the fed state (Figures 3B, D, E) compared to the non-light activated controls. However, there was a strong and complete reversal effect in the food-deprived state (Figure 3C), where *Trh* ∩ *tsh* significantly increased sip durations (Figures 3C, F) and reduced intervals (Figures 3C, G). Our data suggest the enhanced feeding effect obtained in *Trh* ∩ *tsh* flies might be from selective activity in SEL neurons in addition to the effects from VNC serotonergic activity. Collectively, these results highlight that the serotonergic modulation of feeding is primarily from the brain serotonin with some indirect effects from VNC.

### 3.4. Optogenetic inactivation of broad serotonergic neurons enhances feeding

**FIGURE 4:**
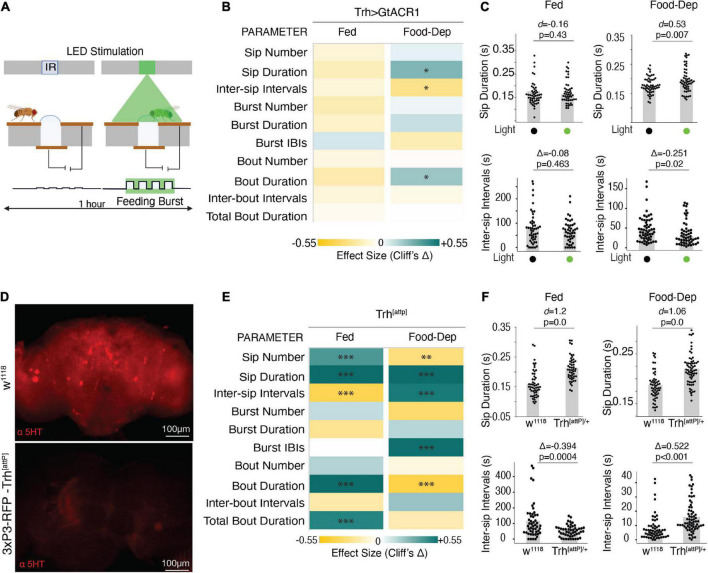
The inactive serotonergic system enhances feeding. **(A)** Schematic depicting the closed-loop OptoPAD system used for optogenetic inactivation of serotonergic neurons as the fly starts to feed. The green illumination lasts for 2 s after the start of the activity bout. **(B)** Heat maps showing Cliff’s Δ effect sizes for all the parameters measured by the OptoPAD system for *Trh-Gal4* > *UAS-GtACR1* with and without green illumination for the fed state and the food-deprived state. **(C)** Cumming estimation plots showing Cohen’s *d* for mean sip durations and Cliff’s Δ for inter-sip intervals of *Trh-Gal4* > *UAS-GtACR1* flies in the fed state (left) and food-deprived state (right) with and without optogenetic activation during the assay time (3,600 s). Inactivation of Trh neurons at 100 uW/mm^2^ increased the sip duration [*n* = 54–55, *d* = 0.53, (95% CI 0.15, 0.89), *p* = 0.0074] and decreased the inter-sip intervals [*n* = 52–57, Δ = –0.265 (95.0% CI –0.46, –0.03), *p* = 0.018] in the food-deprived state. **(D)** Maximum intensity projections of anti-5-HT staining of fly brains with normal expression of Trh (wild-type) and flies carrying the attP mutation. **(E)** Heat map showing Cliff’s Δ effect size for the parameters measured by the OptoPAD system for *Trh-attp/*+ compared to *w*^1118^ controls for the fed and food-deprived states. **(F)** Cumming estimation plot showing Cohen’s *d* for mean sip durations and Cliff’s Δ for inter-sip intervals for *Trh-attP/*+ flies compared with *w*^1118^ flies in the fed state (left) and the food-deprived state (right) during the assay time (3,600 s). The Trh mutants showed an increased sip duration [*n* = 50–51, *d* = 1.2, (95% CI 0.72, 1.66), *p* < 0.0001] and a decrease in the inter-sip intervals [*n* = 49–51, Δ = –0.394 (95.0% CI –0.593, –0.16), *p* = 0.0004] in the fed state. The Trh mutants showed an increased sip duration [*n* = 57–65, *d* = 1.06, (95% CI 0.67, 1.42), *p* = 0.0], and the inter-sip intervals [*n* = 57–67, Δ = 0.522 (95.0% CI 0.328, 0.677), *p* < 0.0001] in the food-deprived state. In the heatmap, green indicates an increase in effect size, and yellow indicates a decrease in effect size. *p*-values for the effect size measure are indicated with an asterisk *p < 0.05, ^**^p < 0.01, and ^***^p < 0.001.

To determine the sufficiency and/or necessity of serotonin for the modulation of these parameters during feeding, we silenced the same neurons representing the broad serotonergic activity in the fly brain using GtACR1 (Mohammad et al., 2017)–an optogenetic inhibitor of neuronal activity upon illumination with green light as the animal starts feeding (Figure 4A). By driving expression of GtACR1 under the control of the Trh-Gal4 promoter, green illumination at a lower intensity of 20 μW/mm^2^ did not show any significant effect on the feeding parameters using the OptoPAD (Supplementary Figure 2). However, in the food-deprived state, the green illumination at 100 μW/mm^2^, flies showed a significantly increased sip duration (Figures 4B, C) with reduced intervals between sips (Figures 4B, C), leading to an overall increase in the food interaction time (Figure 4B) compared to non-light activated controls. In the fed state, however, there was no significant difference in any of these parameters at either light illumination (Figures 4B, C and Supplementary Figure 2). This increase in feeding by acute inactivation of serotonergic neurons in the brain when the animal starts feeding suggests the necessity of serotonin for capping the food intake in a hungry animal.

To further test our hypothesis, we used *Trh*^[attP]^ mutants which carry an insertion mutation in the *Trh* gene and are chronically deprived of serotonin (Figure 4D). We analyzed the feeding behavior of these flies using the FlyPAD and compared it with the wild-type isogenic *w*^1118^ flies. Flies carrying the heterozygous mutation showed a significant increase in feeding in general but with differential modulation of these feeding parameters in a brain state-dependent manner. In the fed state, the flies had a significant increase in the sip number and duration, reduced intervals between the sips, and increased mean duration and total duration of the food interaction time (Figures 4E, F), all consistent with an increase in feeding compared to the wild-type flies, despite being in a fed state.

In the food-deprived state, the mutant flies also showed increased sip duration compared to the wild-type flies (Figures 4E, F). However, there was a decrease in the total number of sips and an increase in the intervals between sips and feeding bursts (Figures 4E, F) which led to an overall decrease in the average duration of the activity bout (Figure 4E). The inconsistencies in the different parameters could be due to the chronic nature of neuronal manipulation exacerbated by the stress of prolonged food deprivation. Despite the differences, our data from both the acute and chronic inactivation of serotonin suggests the necessity of serotonin for controlling food intake at the level of the sips.

### 3.5. Distinct serotonin receptors enhance or suppress feeding

**FIGURE 5:**
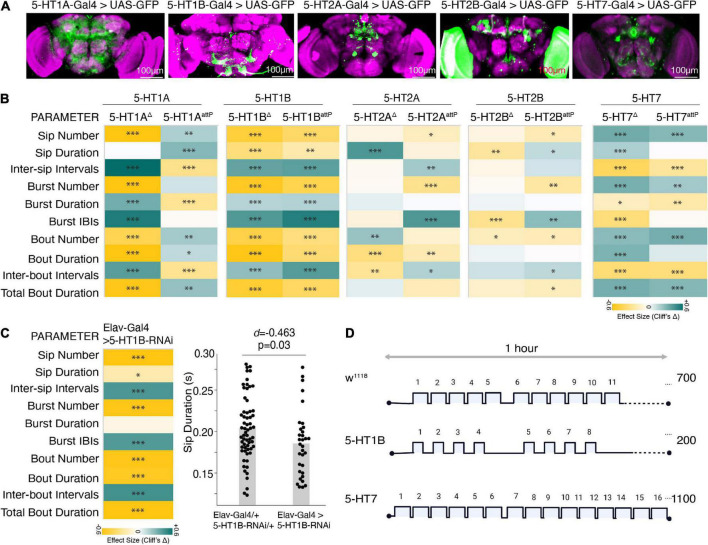
Distinct 5HT receptors enhance or suppress feeding. **(A)** Maximum intensity projections of Gal4 driven 5-HT receptors in the brain expressing mcD8:GFP and immunostained with anti-Dlg, a neuropil labeling antibody. Scale bar is 100 um. **(B)** Heatmap showing Cliff’s Δ effect sizes for changes between the receptor mutants and wild-type flies (as shared control) for all the parameters measured by the FlyPAD. **(C)** Heatmap shows the effects as changes in feeding parameters (Cliff’s Δ) between Elav-Gal4 driven 5-HT1B-RNAi compared to its genotypic controls (Elav-Gal4/+ and UAS-5-HT1B-RNAi/+ combined) and estimation plot showing the reduction in sip duration upon RNA interference. [*d* = –0.463, (95%CI –0.82, –0.1), *p* = 0.03]. **(D)** Representative schematic comparing the changes in feeding microstructure parameters with the receptor mutants compared to wild-type (average number of sips are shown, sip durations and intervals are indicative of changes, not drawn to scale). In the heatmap, green indicates an increase in effect size, and yellow indicates a decrease in effect size. *p*-values for the effect size measure are indicated with an asterisk *p < 0.05, ^**^p < 0.01, and ^***^p < 0.001.

Similar to vertebrates, in *Drosophila*, 5-HT acts through five G-protein coupled receptors–5-HT1A, 5-HT1B, 5-HT2A, 5-HT2B, and 5-HT7 (Saudou and Hen, 1994). These serotonin receptors are variably expressed in the different brain regions (Figure 5A) and can mediate excitatory and inhibitory functions. The 5-HT1A and 5-HT1B are Gi/o coupled receptors that decrease cAMP levels on ligand binding and inhibit neuronal firing (Nichols and Nichols, 2008). On the other hand, the 5-HT2 class and 5-HT7 are Gq and Gs-coupled receptors, respectively, that act through different secondary messengers and increase intracellular calcium, thereby stimulating neuronal firing.

To characterize the effect of 5-HT receptors on feeding microstructure, we used deletion and insertion mutants (details provided in the “Section 2 Materials and methods”) of the five different 5-HT receptors. All the receptor mutants except for 5-HT1B are homozygous lethal; hence the heterozygous F1 progeny of 5-HT receptor mutant flies crossed with *w*^1118^ isogenic flies were assayed on the FlyPAD and compared with age-matched *w*^1118^ isogenic controls. Since the broad serotonin activation had the most consistent and sufficient effect on the feeding parameters in the food-deprived state, we show the results of the FlyPAD assay for the receptor mutants only in the food-deprived state.

All the receptor alleles showed inconsistent effects on feeding parameters, except the 5-HT1B allele in all feeding parameters and the 5-HT7 receptor mutant allele in most parameters. While the heterozygous 5-HT1B receptor mutants showed a marked reduction in all the feeding events with a significant decrease in the feeding rate (decreased sip duration, increased inter-sip intervals, inter-burst, and inter-bout intervals) compared to wild-type flies (Figures 5B–D), heterozygous 5-HT7 receptor mutants showed a significant increase in the feeding rate (increased sip numbers and decreased inter-sip, inter-burst, and inter-bout intervals) compared to wild-type flies (Figures 5B–D). Overall, 5-HT1B and 5-HT7 receptors’ inactivation affect phenocopies Trh activation and inactivation effects, respectively.

As a confirmation, we also knocked down the 5-HT1B receptor using an RNAi line. We found that the effect on feeding parameters remained mostly the same, albeit with small differences in the effect sizes (Figure 5E). Overall, these two receptors, 5-HT1B and 5-HT7, have a marked effect on the feeding microstructure reflecting a decrease and increase in feeding behavior (Figure 5F).

## 4. Discussion

### 4.1. Differences in feeding microstructure in a freely feeding sated and food-deprived state

Hunger induces many behavioral and physiological changes in all animals. An animal’s response to various stimuli can be either attractive or aversive based on its hunger state. The most intuitive change in the behavior of a food-deprived animal would be increased food intake. Adult *Drosophila* eats intermittently and in minuscule amounts, the quantification of which has been a challenging endeavor in fly-feeding research. With the advancement of automated techniques in recent years, several high throughput techniques have been developed which rely on indirect measurement by changes in electrical properties upon food contact. The FlyPAD is one of the available techniques that gives a wealth of information about the microstructure of these fly-feeding events with high temporal resolution. Using this system, we observed that wild-type flies, when food-deprived for a long duration (24 h), ingest a lot more food by having more interactions with the food, many more meals, and each meal consisting of more and longer sips than their fed counterparts. However, the significant reduction in the duration of each meal suggests that when a food-deprived animal gains access to palatable food, it may resort to an adaptive strategy to have many meals but of a shorter duration. This may be to minimize predation risk, heightened anxiety, or a strategy to sample more variable food sources.

### 4.2. Serotonin function in feeding modulation

Several insect species show a decrease in feeding with increasing neuronal or hemolymph serotonin levels (Dacks et al., 2003; Falibene et al., 2012). However, the opposite effect is seen in many other insects like mosquitoes and annelids. For example, the pharmacological depletion of serotonin in mosquitoes led to decreased blood feeding (Novak and Rowley, 1994), and exogenous application of serotonin led to increased blood-feeding in medicinal leeches (Lent and Dickinson, 1984).

In *Drosophila*, thermogenetic activation of serotonin cells has previously been shown to decrease locomotion, mating, and feeding (Pooryasin and Fiala, 2015). Supporting earlier findings, our results indicate that serotonin suppresses feeding in the fed and food-deprived conditions. However, we observed differences at the microstructure level in a brain-state-dependent manner. In fed flies, while serotonin reduced the number of sips and increased intervals, the duration of the sips was similar to that of controls. Hence, the difference in the average duration of each food interaction time wasn’t statistically significant, although the total duration of food interaction was reduced. However, food-deprived flies, despite their strong motivation to feed, significantly reduced the duration of their sips and increased the intervals between the sips compared to their controls, and this remained consistent between replicates.

A previous study has shown that thermogenetic activation of serotonergic neurons shifts the sugar responsiveness of the flies toward higher sugar concentrations (Pooryasin and Fiala, 2015). Our results from the optogenetic PER assay partially agree with previous observations. In our experiments, flies with optogenetically activated serotonin responded similarly to controls when presented with 500 mM sucrose, suggesting no peripheral reduction in gustatory sensory responsiveness at high sucrose concentration. However, in the OptoPAD assay with 500 mM sucrose concentration, the feeding of flies with activated serotonin remained significantly inhibited, with shorter sips and longers intervals between them, confirming earlier observations that serotonin induces behavioral quiescence (Pooryasin and Fiala, 2015). Similar observations have also been shown in other insect species, such as honeybees (French et al., 2014) and ants (Falibene et al., 2012). The slight difference in our data on PER response and earlier studies could also be related to differences in neural actuators (thermogenetics vs. optogenetics) or gender (male vs. female).

Interestingly, the flies show an impaired PER response at lower sucrose concentrations of 5 mM and even with water, which may be either related to the motor deficits induced by serotonin; or is a startling effect of the first light exposure (flies were kept in the dark for retinal treatment). Alternatively, these differences between PER and OptoPAD response could also be because of differences in brain states of freely moving flies compared to tethered flies (Gowda et al., 2022). Serotonin, a psychoactive neurotransmitter, may coordinate the interplay between feeding and stress differently in an immobilized fly.

### 4.3. Serotonin locomotor neurons influence serotonin-mediated feeding in a hunger state-dependant manner

Despite several studies, whether and how serotonin promotes or suppresses feeding and whether locomotor effects of serotonin could affect feeding has yet to be resolved (Tierney, 2020). This is especially true when most serotonin neurons in CNS are manipulated, as serotonin in VNC is known to regulate locomotion (Howard et al., 2019). Given the rhythmic nature of *Drosophila* feeding and its modulation through the sensory feedback system, sensory serotonergic neurons may modulate central pattern generators in the subesophageal zone (Itskov et al., 2014) or VNC (Huckesfeld et al., 2015) to affect feeding microstructure.

While activating most serotonergic cells in CNS is also known to suppress feeding, a smaller subset of serotonergic brain cells have been shown to promote feeding in the sated flies (Albin et al., 2015); interestingly, this subset labeled by R50H05-Gal4 does not express in VNC, a locomotor controlling center. In agreement, our data on activating only brain serotonin cells in sated flies also exhibit enhanced feeding. However, when only VNC serotonin cells were activated during the sated state, it did not affect the feeding microstructure. Overall, our data suggest that during uninduced feeding in sated flies, VNC serotonergic cells on their own don’t affect feeding.

Distinct from the sated state, starvation induces motivation to both eat and move (Knoppein et al., 2000). Given that VNC serotonin has been shown to slow down locomotion (Howard et al., 2019) and lead to suppression of arousability (Pooryasin and Fiala, 2015) or enhancement of immobility (Gowda et al., 2022), activating the locomotor serotonergic circuit in the food-deprived state may have an indirect effect on feeding by inhibiting the locomotor drive mediated by starvation and at the same time promoting feeding by activity in SEL neurons. However, more conclusive experiments, especially imaging VNC in feeding flies and specific serotonergic drivers, are required, which could highlight how VNC affects feeding and how the brain state is involved in this circuit.

### 4.4. Suppressed serotonin system promotes feeding

Serotonin function in modulation of sip duration, and inter-sips intervals in food-deprived flies was further confirmed by the inactivation of the Trh-labeled neurons with GtACR1 and green light illumination, wherein the food-deprived animals showed an increase in sip duration combined with a shorter interval between them compared to their controls. This led to an overall increase in average food interaction time and hence more food intake. This is consistent with a similar approach using thermogenetic inhibition of Trh-Gal4 neurons, which was shown to increase food intake (Eriksson et al., 2017). Our results explain this increased food intake at the level of the temporal aspects of individual sips and the intervals between them.

Similar to mammals, *Drosophila* has a compartmentalized neuronal and peripheral serotonin synthesis. Trh–null flies, which lack the enzyme for the neuronal synthesis of serotonin, showed reduced feeding ability in both larval and adult stages (Neckameyer et al., 2007). Our experiments with Trh^[attp]^ mutants showed diverse results in the fed and food-deprived states. While the fed flies had higher food intake than wild-type controls with more and longer sips and shorter intervals and overall, more interaction with the food. Interestingly, in food-deprived flies, there was indeed a reduction in the number and frequency of sips. However, the increase in sip duration was statistically significant compared to the wild-type flies. This supports our hypothesis that serotonin modulates sip duration in the food-deprived state and that neuronal serotonin is necessary for limiting the duration of sips in food-deprived flies. The developmental defects could explain the reduction in overall feeding observed in these flies, which carry the Trh mutation and are chronically deprived of serotonin, compared to acute serotonergic depletion in the GtACR1 inactivation experiments. Serotonergic inputs on the shaping of feeding circuits have been identified. An inverse relationship was observed between the levels of 5HT during development and the neurogenesis of the feeding circuit in *Drosophila* larvae (Neckameyer, 2010).

### 4.5. Multiple serotonin receptors are involved in feeding-related decisions

In both mammals and invertebrates, serotonin cells are relatively few in number, but it is the extensive axons and a wide array of receptors that allow serotonin to have such widespread effects. Artificial global serotonin activation may show non-specific effects by differential stimulation of receptors, especially if food intake is the only readout (Rodgers et al., 2010). Our analysis of feeding microstructure with the receptor mutants and RNAi knockdown is one step closer to resolving these differences. Inconsistencies in behavior between receptor mutant lines could result from these non-specific effects combined with the chronic nature of these mutations and the complex underlying biology. Hence, we have used two lines of evidence to infer the role of the receptor in the feeding microstructure.

Among these, the 5-HT1B mutation consistently reduced the feeding parameters, particularly the sip duration and increased intervals. Previously, it was shown that blocking 5-HT1B expressing abdominal leucokinin neurons in *Drosophila* leads to increased desiccation resistance and less food intake (Liu et al., 2015). In rodents, the role of 5-HT1B signaling in appetite and food intake has been especially well studied and has been shown to modulate food (fat) intake, wherein blocking these receptors reverses the pharmacologically induced hypophagia (Lin and York, 2005). Also, the 5-HT1B agonist induces anorexia in both freely feeding (Kennett et al., 1987) and food-deprived rats (Bendotti and Samanin, 1987).

In *Drosophila*, the 5-HT2A receptor is also known to play a role in feeding. Blocking the 5-HT2A receptor with metitepine decreases feeding behavior in larvae (Gasque et al., 2013). Starved flies in which Trh neurons were inactivated along with 5-HT2A receptor antagonist Ketanserin showed an aversion to protein food similar to fully fed controls (Ro et al., 2016). In rodents, the 5-HT7 receptors express food motivation and satiety controlling acetylene-releasing neurons; however, using systemic drug studies, they are found not to be regulating food intake (Clissold et al., 2013). However, no other study has analyzed the effects of genetically removed 5-HT7 receptors on feeding in any model system. Our results agree with the known inhibitory and stimulatory nature of 5-HT1B receptors and 5-HT7 receptors, respectively, and suggest that serotonin uses the inhibitory 5-HT1B receptors to stop and excitatory 5-HT7 receptors to promote feeding.

### 4.6. The conclusion and significance of this research

**FIGURE 6:**
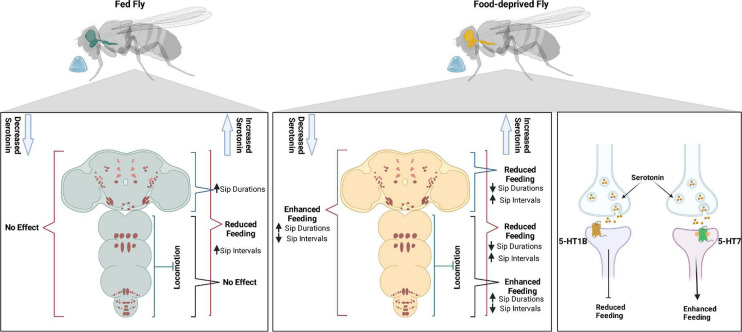
Serotonin modulates feeding in a state-dependant manner using 5-HT1B and 5-HT7 receptors.

Through feeding microstructure analysis, we have shown that food-deprived flies feed with longer sips and sip intervals of shorter duration than sated flies. Optogenetic manipulations of most serotonin cells in sated and food-deprived flies had a major influence on sip duration and inter-sip intervals, suggesting these feeding microstructure parameters to be fine-tuned with serotonergic activity. While agreeing with the previous literature on the overall role of serotonin in suppressing feeding, our study provides novel insight into identifying sip duration and inter-sip intervals as the affected feeding microstructure controlled by serotonin. Using intersection genetics, we have shown the involvement of VNC locomotor neurons either in combination with SEL/PLP neurons or alone affecting feeding behavior in a hunger state-dependent manner. We identified 5-HT1B and 5-HT7 receptors, which serotonin uses to suppress and enhance feeding, respectively (Figure 6).

### 4.7. Limitations of this study

Using *Drosophila*, with its formidable genetic tools and ability to manipulate neural activity, we studied the role of serotonin. However, there are a few limitations in this study; first, our study investigates the flies’ feeding in-depth at the microstructure level. While that provides important insight into feeding patterns, it only indicates food intake and is not an absolute measure. Secondly, we have studied the role of serotonin in sucrose feeding only. Hence, further analyses, such as choice assays between different food sources, would be valuable in elucidating the role of these serotonergic neurons. Thirdly, we have looked at the role of the global activation of serotonin. However, the underlying circuitry could be much more complex, and different subsets of serotonin cells may control feeding behavior distinctly in a network and isolation (Albin et al., 2015).

Lastly, we have measured the feeding behavior only in males; however, given that sex differences have previously been identified feeding choices (Kubli, 2010; Camus et al., 2018) and in response to neuroactive compounds (Sharma et al., 2009) in *Drosophila*, it will be interesting to study how female flies respond to manipulations in serotonergic activity. Driving expression only in certain subsets and analyzing the feeding patterns combined with advanced techniques, such as calcium imaging, could help resolve the role of serotonin further to the level of subclusters, individual cells, and molecules, which will be imperative in understanding global and local effects of serotonin, an important consideration for understanding and developing therapeutic strategies for feeding-related disorders.

## Data availability statement

The original contributions presented in this study are included in the article/Supplementary material, further inquiries can be directed to the corresponding author.

## Author contributions

FM: conceptualization, supervision, project administration, funding acquisition, and instrumentation. AB, SBMG, SS, and FM: methodology. AB: investigation (genetics, FlyPAD and OptoPAD, PER experiments, and neuroanatomy) and data analysis. SBMG: investigation (genetics, neuroanatomy, and assistance to AB in PER experiments). SS: investigation (assistance to AB in FlyPAD and OptoPAD experiments). FM: writing—original draft with contribution from AB. FM writing revision with contribution from AB, SBMG, and SS. FM and AB: visualization. All authors final approval for publication, read, and agree to the published version of the manuscript.

## Footnotes

1
https://flypad.rocks/
