# Supplementary material for: Serotonergic control of feeding microstructure in Drosophila
Source: Front Behav Neurosci. 2023 Jan 17;16:1105579. doi: 10.3389/fnbeh.2022.1105579 (PMC9887136; doi:10.3389/fnbeh.2022.1105579)
Supplement: Supplementary file 1 [file Data_Sheet_1.docx]

# **Supplementary Figures**


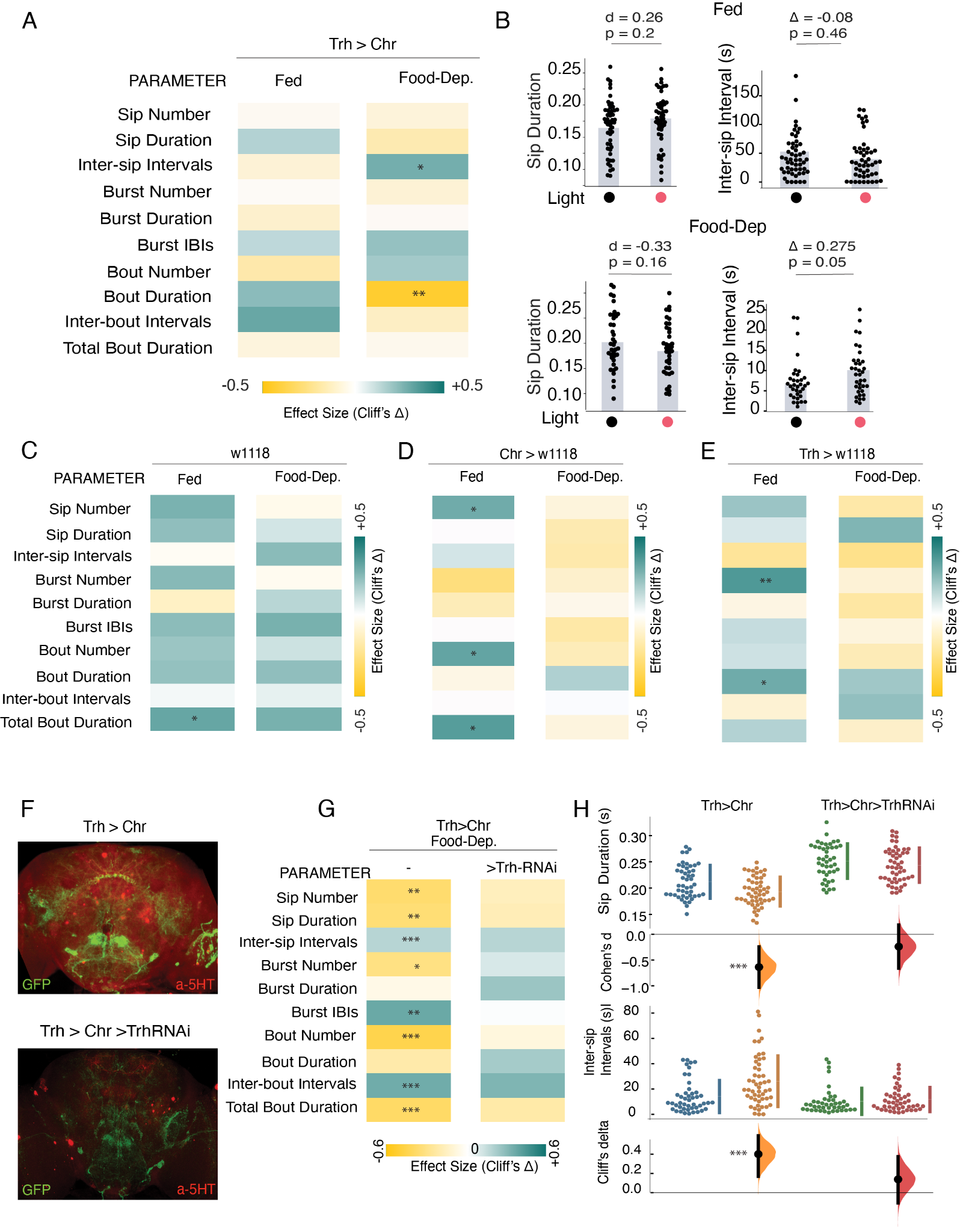


**Supplementary *Figure S1 (related to Fig 2)***

*A* Heat map showing Cliff’s delta effect size for all the parameters measured by the optoPAD system for Trh>Chr in the fed (left) and the food-deprived state (right) at lower red illumination of 35 μW/mm^2^ compared with non-light activated controls.

B. Estimation plots of the sip duration and inter-sip intervals for Trh>Chr in the fed and the food-deprived state at red illumination of 35 μW/mm^2^ illumination compared with non-light activated controls.

C. Heat map showing Cliff’s Δ effect size for all the parameters measured by the optoPAD system for w1118 in the fed (left) and the food-deprived state (right). Flies with red illumination of 100 μW/mm^2^ are compared with non-light activated controls in each state.

D. Heat map showing Cliff’s Δ effect size for all the parameters measured by the optoPAD system for *UAS-Chrimson>w1118* in the fed (left) and the food-deprived state (right). Flies with red illumination of 100 μW/mm^2^ are compared with non-light activated controls in each state.

E. Heat map showing Cliff’s Δ effect size for all the parameters measured by the optoPAD system for *Trh-Gal4>w1118* in the fed (left) and the food-deprived state (right). Flies with red illumination of 100 μW/mm^2^ are compared with non-light activated controls in each state.

F. Maximum intensity projections of Trh-Gal4 expression of UAS-Chrimson-YFP which marks the neuronal projections, immunostained with anti-5HT antibody that marks the serotonergic cell bodies. The scale bar is 100 um.

G. Heat map showing Cliff’s Δ effect size for the 10 parameters measured by the optoPAD for Trh>Chr, Trh>Chr; Trh-RNAi with and without red illumination in the food deprived state.

H. Cumming estimation plot showing the effect of light activation on sip duration and inter-sip intervals in Trh>Chr and Trh>Chr;Trh-RNAi flies.


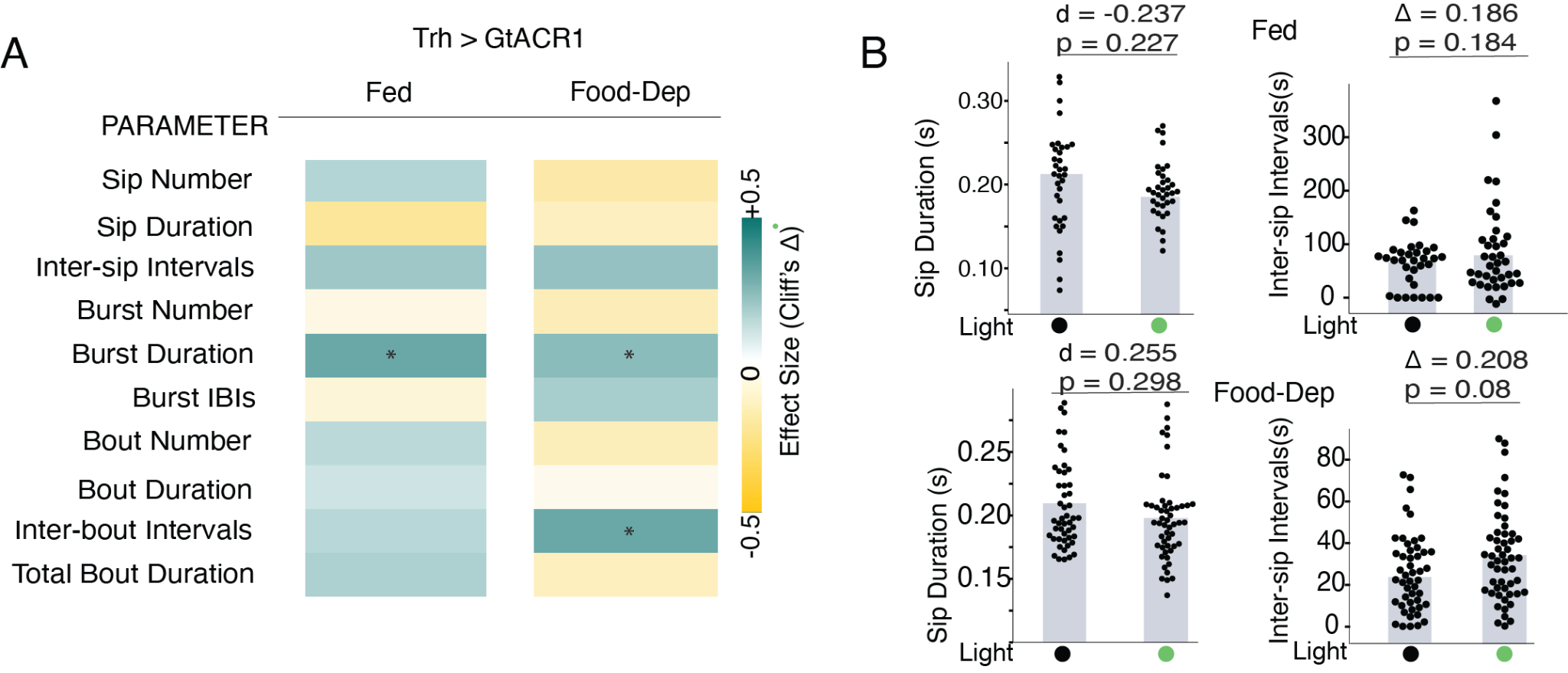


**Supplementary *Figure S2 (Related to fig 4)***

1. Heat map showing Cliff’s Δ effect size for all the parameters measured by the optoPAD system for *Trh-Gal4>UAS-GtACR1* in the fed (left) and the food-deprived state (right). Flies with green illumination of 20 μW/mm^2^ are compared with non-light activated controls in each state.
2. Estimation plots of the sip duration and inter-sip intervals for*Trh-Gal4>UAS-GtACR1*  in the fed and the food-deprived state at red illumination of 35 μW/mm^2^ illumination compared with non-light activated controls.

*
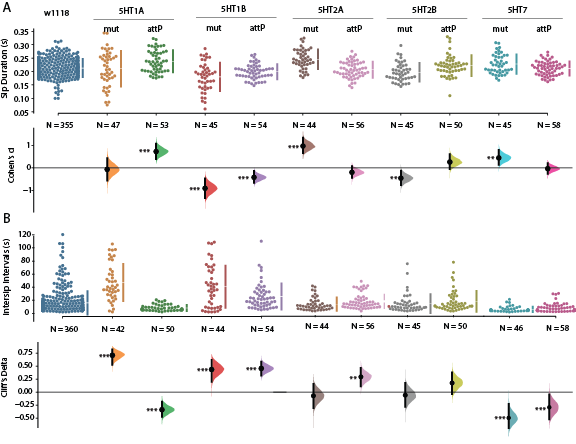
*

**Supplementary *Figure S3 (Related to fig 5)***

A. Cumming estimation plot showing the Cohen's *d* for sip durations of the two types of mutants for the five receptor mutants compared against the shared control w1118 during the assay (3600 sec). A consistent decrease in inter-sip intervals was seen for both the mutant types for 5HT1B  *(w1118; 5HT1B*^Δ^ *(n=45, d=-0.902 [95%CI -1.33, -0.48], p<0.0001) (w1118; 5HT1B^attP^, n=54, d=-0.416 [95%CI -0.66, -0.136 ], p=0.006)*.

B. Cumming estimation plot showing Cliff’s Δ for effect on inter-sip intervals of the two types of mutants for each of the 5 receptors compared against the shared control *w1118* during the assay (3600 sec). A consistent increase in inter-sip intervals was seen for both the mutant types for 5HT1B *(w1118;5HT1B*^Δ^*,* *n=44, Δ=0.434, [95%CI 0.198, 0.618], p<0.001)* *(w1118;5HT1B^attP^, n=54, Δ=0.454, [95%CI 0.32, 0.58], p<0.001)*. A consistent decrease in inter-sip intervals was seen for both the mutant types for 5HT7 *(w1118;5HT7*^Δ^*, n=46, Δ=-0.47, [95%CI -0.59, -0.298], p<0.001) (w1118;5HT7^attP^, n=58, Δ=-0.29, [95%CI,-0.44, -0.13], p=0.0008)*

# **Supplementary Tables**

**Supplementary table 1. Strains of *D. melanogaster* used in this study.**

| **Fly Strain** | **Source** |
| --- | --- |
| **Wild-type** | |
| iso^w1118^ | RRID:BDSC #5905 |
| **Gal4 Stocks** | |
| Trh Gal4 (III) | RRID:BDSC # 38389 |
| 5-HT1A-Gal4 (II) | RRID:BDSC #84588 |
| 5-HT1B-Gal4 (II) | RRID:BDSC #86276 |
| 5-HT2A-Gal4 | RRID:BDSC #86277 |
| 5-HT2B-Gal4 | RRID:BDSC #84445 |
| 5-HT7-Gal4(III) | RRID:BDSC #86279 |
| **UAS Stocks** | |
| 20X-UAS-Chrimson::EYFP (X) | RRID:BDSC #55134 |
| 20X-UAS-GtACR1::EYFP (III) | N/A |
| 20X-UAS-hexameric GFP | RRID:BDSC #52262 |
| 20X-UAS-mcD8-GFP | RRID:BDSC#32185 |
| **Gal 80 Stocks** | |
| *Tsh-Gal80; MKRS/Tb* | N/A |
| *Tsh-Gal80; UAS- Chrimson/Tb* | N/A |
| *Tub<Gal80>; Tsh- LexA, LexA-OP, Flp; Chrimson/Tb* | N/A |
| **RNAi Stocks** | |
| *Trh-RNAi1 (A+ B) RNAi* | N/A |
| 5-HT1B RNAi | RRID:VDRC #v9558 |
| 5-HT1A^[attp]^ | RRID:BDSC #84704 |
| 5-HT1B^[attp]^ | RRID:BDSC #84443 |
| 5-HT2A^[attp]^ | RRID:BDSC #84444 |
| 5-HT2B^[attp]^ | RRID:BDSC #84445 |
| 5-HT7^[attp]^ | RRID:BDSC #84446 |
| 5-HT1A^Δ5kb^ | RRID:BDSC #27640 |
| 5-HT1B^[III-V]^ | RRID:BDSC #55846 |
| 5-HT2A^[c1644]^ | RRID:BDSC #4830 |
| 5-HT2B^[52080]^ | RRID:BDSC #42994 |
| 5-HT7^(f05214)^ | RRID:BDSC #18848 |

**Supplementary table 2. Fly genotypes with their associated figures.**

| **EXPERIMENTAL LINE GENOTYPE** | **ASSOCIATED FIGURE** |
| --- | --- |
| *Iso^w1118^* | Fig 1B-1G, Fig S1D, Fig 4D, Fig 5B, Fig S3A & S3B |
| *+/Y ; +/+; Trh-Gal4/UAS-10X GFP* | Fig 2B, Fig S1A, Fig S1G |
| *UAS-Chrimson/Y ; +/+; Trh-Gal4 /+* | Fig 2C – 2I, Fig 3A – 3G, Fig S1H & S1I |
| *UAS-Chrimson/Y ; +/+;+/+* | Fig S1E |
| *+/Y ; +/+; Trh-Gal4 /+* | Fig S1F |
| *+/Y ; Trh-RNAi1/+;Trh-Gal4 /UAS-Chrimson* | Fig S1G – Fig S1I |
| *+/Y ; tsh-Gal80/+; Trh-Gal4 /UAS-Chrimson* | Fig 3A – 3G |
| *tub>gal80>/Y;tsh-LexA,LexAop-Flp/+; Trh-Gal4/UAS-Chrimson* | Fig 3A – 3G |
| *+/Y;+/+;Trh-Gal4 /UAS-GtACR1* | Fig 4A – 4C, Fig S2A & S2B |
| *3xP3-RFp-Trh^[attP]^* | Fig 4D |
| *+/Y;+/+;Trh^[attP]^/+* | Fig 4E and 4F |
| *+/Y; 5-HT1A-Gal4/+;20X-UAS-hexameric GFP/+* | Fig 5A |
| *+/Y; 5-HT1B-Gal4/+;20X-UAS-hexameric GFP/+* | Fig 5A |
| *+/Y; +/+; 5-HT2A-Gal4/20X-UAS-hexameric GFP* | Fig 5A |
| *+/Y; +/+; 5-HT2B-Gal4/20X-UAS-hexameric GFP* | Fig 5A |
| *+/Y; +/+; 5-HT7-Gal4/20X-UAS-hexameric GFP* | Fig 5A |
| *+/Y; 5-HT1A^[attp]^ /w^1118^ ;+/+* | Fig 5B, Fig S3A & S3B |
| *+/Y; 5-HT1B^[attp]^ /w^1118^ ;+/+* | Fig 5B, Fig S3A & S3B |
| *+/Y; +/+; 5-HT2A^[attp]^ /w^1118^* | Fig 5B, Fig S3A & S3B |
| *+/Y; +/+;5-HT2B^[attp]^ /w^1118^* | Fig 5B, Fig S3A & S3B |
| *+/Y; +/+;5-HT7^[attp]^ /w^1118^* | Fig 5B, Fig S3A & S3B |
| *+/Y; 5-HT1A*^Δ^ */w^1118^ ;+/+* | Fig 5B, Fig S3A & S3B |
| *+/Y; 5-HT1B*^Δ^ */w^1118^ ;+/+* | Fig 5B, Fig S3A & S3B |
| *+/Y; +/+; 5-HT2A*^Δ^*/w^1118^* | Fig 5B, Fig S3A & S3B |
| *+/Y; +/+;5-HT2B*^Δ^ */w^1118^* | Fig 5B, Fig S3A & S3B |
| *+/Y; +/+;5-HT7*^Δ^*/w^1118^* | Fig 5B, Fig S3A & S3B |
| *+/Y; Elav-Gal4/+ ; 5HT1B-RNAi / +* | Fig 5C |
| *+/Y; Elav-Gal4/+ ; + / +* | Fig 5C |
| *+/Y; +/+ ; 5HT1B-RNAi / +* | Fig 5C |
